# A lossless compression method for multi-component medical images based on big data mining

**DOI:** 10.1038/s41598-021-91920-x

**Published:** 2021-06-11

**Authors:** Gangtao Xin, Pingyi Fan

**Affiliations:** The Department of Electronic Engineering, Tsinghua University, Beijing, 100084 China

**Keywords:** Applied mathematics, Information technology, Statistics, Electrical and electronic engineering

## Abstract

In disease diagnosis, medical image plays an important part. Its lossless compression is pretty critical, which directly determines the requirement of local storage space and communication bandwidth of remote medical systems, so as to help the diagnosis and treatment of patients. There are two extraordinary properties related to medical images: lossless and similarity. How to take advantage of these two properties to reduce the information needed to represent an image is the key point of compression. In this paper, we employ the big data mining to set up the image codebook. That is, to find the basic components of images. We propose a soft compression algorithm for multi-component medical images, which can exactly reflect the fundamental structure of images. A general representation framework for image compression is also put forward and the results indicate that our developed soft compression algorithm can outperform the popular benchmarks PNG and JPEG2000 in terms of compression ratio.

## Introduction

Resources are always limited. Whether storage space or communication bandwidth, is not usually sufficient, which inspires us to apply compression that aims to reduce the number of bits needed to represent an entity to meet the actual situation. In most telemedicine applications, the role of image compression techniques is significant to deal with the medical images^1^. Image compression greatly reduces the space required for storage and the bandwidth needed for transmission, which is not only conducive to the widespread use of medical imaging, but also one of the most important basic technologies of intelligent medicine. For medical images, there are two extraordinary properties, giving the starting point of image compression technology.

① **Lossless** There are two categories of image compression: lossy compression and lossless compression^2^. The image reconstructed by lossy compression is visually similar to the original image, but not absolutely the same. On the other hand, the image reconstructed by lossless compression is exactly the same as the original image. In medical imaging, we generally do not adopt lossy compression as it may cause the loss of critical information in an image, which would lead to the doctor’s misjudgment of the disease^3^.

② **Similarity** It includes both mutual similarity and self similarity. The scanning images of the same part of different people are similar, such as lungs, kidneys, eyes, etc. They are similar in general, but different in detail. When scanning separate parts of the same person, there are also similarities, such as continuous scanning of blood vessels or skin.

For medical image compression, in addition to the classical methods, such as Huffman coding^4^, arithmetic coding^5^, Golomb coding^6^, Run length coding^7^, LZW coding^8^, predictive coding^9^ and so on, there are also some novel algorithms.

The widely used JPEG^10^ and JPEG-2000^11^ were based on discrete cosine transform^12^ and wavelet transform^13^, respectively. The method based on transform domain is also a large class of medical image compression. In^14^ and^15^, they were based on discrete cosine-based discrete orthogonal stockwell transform^16^ and integer wavelet transform, respectively. In^17^, it was based on Burrows-Wheeler transformation with an inversion coder^18^. In^19^, it adopted incremental self organizing map and discrete wavelet transform. In addition, there are also some methods^20^ that focused on the selection of wavelet for compression of medical images. All of these methods transform the spatial domain to another domain, thus processing the transformed coefficients.

Because of the situation that some medical images only focus on the region of interest^21^, applying lossless compression to the region of interest and lossy compression to the region of non interest becomes a solution^22^. As a part of the region of interest, in^3^, it proposed an algorithm using discrete wavelet transform and set partitioning in hierarchical trees algorithm, which aims to eradicate the noisy content in the background and resurrect the positions of medical image in lossless manner. In^23^ and^24^, they used the LZW technique and extended bit depth for compressing the medical images by finding the region of interest on an image, respectively. In^22^, it attempted to implement the region of interest based image compression using embedded zero-tree wavelet algorithm for medical images. In^25^, it adopted a context-based and region of interest based approach to compress medical images in particular vascular images. Similarly, in^26^, it considered a multi-region of interest medical image compression problem with edge feature preserving.

The compression method based on prediction can make good use of the continuity of an image^27,28^. In^29^, it proposed an method for the compression of medical images that exploits the three-dimensional nature of the data by using linear prediction. The paper^30^ proposed a lossless compression scheme based on prediction by partial matching. In^31^, it adopted a method that combines super-spatial structure prediction with interframe coding to achieve compression effect. There are also some algorithms not specially designed for medical image compression^32–35^, which can be applied due to their generality. The image compression methods based on neural network^36,37^introduce the concept of learning into this filed, possessing good performance. Perceptually lossless compression^38,39^ can attain higher compression performance without loss of important information and has good application potential over bandwidth limited channels.

Using the characteristics of medical images to complete compression is the mainstream direction. Due to the fact that mostly the human body possesses bilateral symmetry, which means that an organ of the human body can be divided into two symmetrical halves by simply drawing a vertical line down their centers. In^40^, it proposed an approach to compressing the medical images by making use of their symmetry feature. The paper^41^ represented a hybrid lossless compression method that combines a segmentation technique with a lossless compression scheme. There are also some methods that combine these technologies to apply to medical image compression. The paper^42^ adopted HEVC for diagnostically acceptable medical image compression. In^43^, it proposed a method in medical image compression by using sequential-storage of differences technique.

For an image, it is a combination of numerous pixels, which not only contains the intensity value of each pixel, but also includes its location. However, in conventional image representation methods, pixel intensity values are stored in a certain order (such as scanning from left to right and from top to bottom). These approaches turn the location into a definite quantity, which leads to no need to encode the location. In fact, the method of not considering the intensity value of each pixel and its location at the same time is certainly not as good as that of the compression ratio from both directions simultaneously.

**Figure 1:**
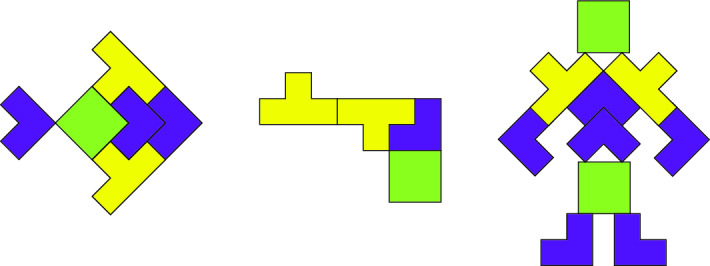
Some patterns (including fish, pistol and robot) formed by the combination of basic shapes.

**Figure 2:**
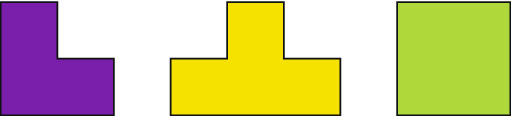
Some basic shapes can be arranged and combined into patterns.

Let us see a toy model. Figure 1 illustrates three patterns: fish, pistol and robot, but they are all only made up of the three basic shapes shown in Fig. 2. When we see these two figures, we will think that if these shapes in Fig. 2 are used as the basic units of the image to complete the compression of several patterns in Fig. 1, the compression effect will be great. Data mining^44,45^ points out the way to solve this problem.

In recent years, data mining is one of the most interesting area of research that includes classification, clustering, association, regression in health domain^46^. In^47^, it presented a ransription factors network in the major organs of the mouse, allowing data mining and generating knowledge to elucidata the roles in various biological processes. The paper^48^ combined predictive data mining with experimental evaluation in patient-derived xenograft cells to identify therapeutic options for high-risk patients. In^49^, data mining and model-predicting were used in a global disease reservoir for low-pathogenic avian influenza.

Our purpose is to find the basic shape in images similar to Fig. 2 with data mining. This is exactly the starting point of soft compression whose basic component unit is the shape, representing an image by using both shapes and locations. Of course, this is merely a visual explanation for soft compression. The actual algorithm is more scientific and theoretical than this example. Soft compression is a lossless image compression method whose codebook is no longer designed artificially or only through statistical models but through data mining, which can eliminate coding redundancy and spatial redundancy simultaneously. It was first proposed in^50^, dedicated to binary image compression. Then in^51^, soft compression was analyzed theoretically and the compression algorithm for gray image is designed.

In this paper, we present a general framework for representing image compression in philosophy. Under the guidance of this framework, a new multi-component image compression algorithm based on big data mining is designed, which is especially serviceable for medical images.

## Results

### A general representation framework for image compression

In this new framework, we adopt the basic unit instead of the pixel as the component unit of an image. It provides a point of view to consider both coding redundancy and spatial redundancy simultaneously. The basic unit can be pixel intensity values one by one, or shapes and symbols combined by different pixels.

Let *I* denote a digital image which is composed of a great deal of basic units, whose row and column dimensions are *M* and *N*, respectively. Let $l_I(x_i,y_i)$ and $l_P(x_i,y_i)$ represent the number of bits needed to denote a basic unit and its location, then the number of bits required for an image is

1 $$\begin{aligned} B=\sum _{i=1}^T [l_I(x_i,y_i)+l_P(x_i,y_i)] \end{aligned}$$

where *T* is the number of basic units needed to represent an image.

For Huffman coding^4^, the storage order is in a certain mode, so only the probability distribution of pixel intensity value is considered. The location is not taken into account, so $l_P(x_i,y_i)$ becomes zero (Because the location loses randomness and becomes a certain quantity when it is encoded in a certain order, namely, the entropy of the location is zero). The basic unit with huffman coding for images is a single pixel, so $T=MN$, formula (1) can be simplified as (2).

2 $$\begin{aligned} B_{hf} = \sum _{i=1}^{MN} [l_I(x_i,y_i)] \end{aligned}$$

The representation of Golomb coding^6^ is the same as huffman coding, as shown in formula (3). The difference is that Golomb coding is designed for non-negative integer input with geometric distribution.

3 $$\begin{aligned} B_{golomb} = \sum _{i=1}^{MN}[l_I(x_i,y_i)] \end{aligned}$$

LZW coding^8^ is also stored in a certain mode and $l_P(x_i,y_i)=0$. In this method, the number of basic units is not *MN*, but a value *T* less than *MN*, as shown in (4).

4 $$\begin{aligned} B_{lzw} = \sum _{i=1}^T[l_I(x_i,y_i)] \end{aligned}$$

Run length coding^7^ compresses the repeated symbols, and uses a fixed number of bits to represent the number of repetitions of the symbol. In this way, formula (1) can be expressed as (5).

5 $$\begin{aligned} B_{rl} = \sum _{i=1}^T[l_I(x_i,y_i)+l_C] \end{aligned}$$

where $l_C$ is the required number of bits to represent a location.

Symbol-Based coding^52^ is mainly designed for document storage, which takes the repeated characters in the text as a symbol. It considers both symbols and locations, which can be expressed by formula (6).

6 $$\begin{aligned} B_{sb}=\sum _{i=1}^T[l_I(x_i,y_i)+l_P(x_i,y_i)] \end{aligned}$$

The representation of soft compression is similar to Symbol-Based coding and can be expressed by formula (7). The difference is that the basic unit of soft compression is the shape, which is obtained by searing in datasets based on data mining rather than by artificial design. Compared with Symbol-Based coding, it is more close to the nature of images and reflects the essential information of a dataset.

7 $$\begin{aligned} B_{sc} = \sum _{i=1}^T [l_I(x_i,y_i)+l_P(x_i,y_i)] \end{aligned}$$

In this new framework, we can unify the representation of different compression methods, which is helpful to the comparison and analysis of diverse approaches. We summarize these methods in Table 1.

**Table 1:** Some image compression methods.

Method	Basic unit	Number	Bits required per location
Huffman coding	Pixel	$M\times N$	0
Golomb coding	Pixel	$M\times N$	0
LZW coding	Pixel and their combination	*T*	0
Run length coding	Pixel	*T*	$l_C$
Symbol-Based coding	Symbol	*T*	$l_P(x_i,y_i)$
Soft compression	Shape	*T*	$l_P(x_i,y_i)$

#### Soft compression algorithm for multi-component image

For a multi-component image, it is first decomposed into multiple single component images and then reversible component transformation is performed. Each single component image will be divided into the shape layer and detail layer after predictive coding and mapping. The shape layer is regular and sparse, while the detail layer is irregular and dense. Therefore, different layers should be coded depending on their properties. The compressed image can be obtained by combining the coding data of each shape layer and detail layer of each single component image.

**Figure 3:**
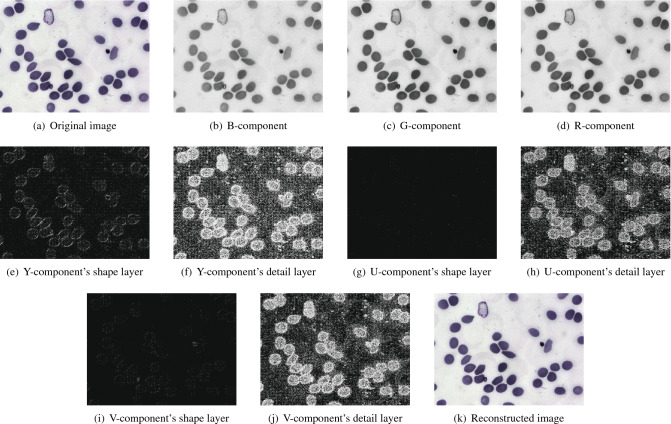
An instance of Malaria dataset by using soft compression algorithm for multi-component image. (**a**) The original RGB image. (**b**) B-component image. (**c**) G-component image. (**d**) R-component image. (**e**) The shape layer of Y-component. (**f**) The detail layer of Y-component. (**g**) The shape layer of U-component. (**h**) The detail layer of U-component. (**i**) The shape layer of V-component. (**j**) The detail layer of V-component (**k**) The reconstructed image by decoding.

Malaria is a disease caused by Plasmodium parasites that remains a major threat to global health, affecting 200 million people and causing 400,000 deaths a year. Identifying and quantifying malaria could have a huge significance for research in both the medical and computer science field, whose dataset^53^ will be employed to reveal sound effects of the soft compression algorithm. Figure 3(a) is a multi-component image from Malaria dataset. We will try to use the visual representation of this image to describe each step in the encoding and decoding process of soft compression algorithm. With regard to a multi-component image, the first step is to decompose it into three single component images B, G and R, as shown in Fig. 3(b), (c) and (d). These three single components represent the intensity of blue, green and red of each pixel in the image. The next step which takes advantage of the correlation between these components is to perform reversible component transformation to generate three new components Y, U and V, namely, color decorrelation for efficient compression, reasonable color space with respect to the human visual system and ability of having lossless compression^11^. The transformation of two color spaces takes the same form as JPEG2000^11^, which is shown in the formula (8).

8 $$\begin{aligned} \begin{aligned} \begin{pmatrix} Y \\ V \\ U \end{pmatrix} = \begin{pmatrix} \left\lfloor \displaystyle {\frac{R+2G+B}{4}}\right\rfloor \\ R-G \\ B-G \end{pmatrix} \qquad \begin{pmatrix} G \\ R \\ B \end{pmatrix} = \begin{pmatrix} Y-\left\lfloor \displaystyle {\frac{U+V}{4}}\right\rfloor \\ V+G \\ U+G \end{pmatrix} \end{aligned} \end{aligned}$$

**Figure 4:**
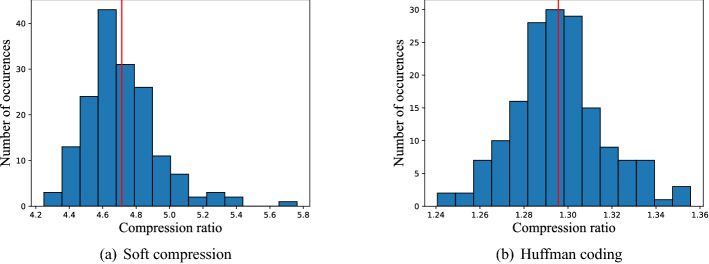
The histogram of compression ratio of BCCD dataset with soft compression algorithm for multi-component image and Huffman coding. (**a**) Soft compression. (**b**) Huffman coding (The compression ratio is defined as the number of bits required for natural binary code divided by another specific coding method when representing the same image).

The Y-component image is processed through multiple processing steps (which will be described in the method section). The shape layer image and detail layer image of Y-component are obtained by layer separation, as shown (binarization has been made for clearer appearance) in Fig. 3(e) and (f). The reason for layering is that different coding methods will be adopted according to the different properties of shape layer and detail layer. The former is regular and sparse, while the latter is irregular and dense. Therefore, for the shape layer, the shape is regarded as the basic unit for representing an image. The number of bits required to represent a shape layer image containing *T* shapes is $\sum _{i=1}^T [l_I(x_i,y_i)+l_P(x_i,y_i)]$, where $l_I(x_i,y_i)$ and $l_P(x_i,y_i)$ represent the length to denote a shape and its location respectively. Due to the irregularity of the detail layer, it can be encoded by common statistical coding methods. Similarly, the other two components are taken with the same treatment as the Y component, which are illustrated from Fig. 3(g) to (j). Decoding the compressed data from Fig. 3 with soft compression algorithm for multi-component image can acquire the reconstructed image, which is shown in Fig. 3(k). The compression ratio of this instance is 4.40, which largely eliminates coding redundancy and spatial redundancy.

BCCD dataset is a small-scale dataset for blood cells detection. We select the first 200 images of the BCCD dataset as the training set and the remaining 166 images as the testing set. Then, soft compression algorithm and traditional Huffman coding are applied to gain the compression ratio, and their results are statistically analyzed to obtain the frequency histogram, Fig. 4(a) and (b). The results of Huffman coding come from the independent coding of the three components without any other processing. The comparison indicates that if an image is compressed only from the perspective of coding redundancy, the results will be poor. From these two figures, we can draw a conclusion that soft compression is much better than traditional Huffman coding in lossless image compression because it aims to eliminate both coding redundancy and spatial redundancy simultaneously.

Table 2 illustrates the experimental results of soft compression algorithm for multi-component images and other classical systems on Malaria, BCCD, Melanoma and FIRE^54^ datasets. The statistics include mean, minimum, maximum and variance about compression ratio. The results of Table 2 indicate that the average compression ratio with soft compression is obviously higher than other image lossless compression methods. Through comparison, we can reach a conclusion that soft compression algorithm for multi-component algorithm outperforms the popular classical benchmarks JPEG, PNG and JPEG2000.

**Table 2:** Some statistics about compression ratio of Malaria, BCCD, Melanoma and FIRE datasets by using different compression methods (JPEG and JPEG2000 are in lossless mode).

Dataset	Statistic	Method			
		Soft compression	JPEG (lossless mode)	PNG	JPEG2000 (lossless mode)
Malaria	Mean	**3**.**80**	2.53	2.83	3.56
	Minimum	2.35	1.77	1.87	2.50
	Maximum	6.88	4.41	7.46	8.92
	Variance	0.8058	0.3401	1.0340	1.2497
BCCD	Mean	**3**.**83**	2.24	2.49	3.56
	Minimum	3.34	2.06	2.18	3.11
	Maximum	4.57	2.54	2.87	4.20
	Variance	0.0535	0.0089	0.0166	0.0369
Melanoma	Mean	**3**.**44**	1.84	1.93	3.15
	Minimum	1.81	1.29	1.31	2.02
	Maximum	5.17	2.81	3.11	4.70
	Variance	0.4758	0.0596	0.0867	0.2365
FIRE	Mean	**4**.**71**	2.69	3.52	4.66
	Minimum	4.23	2.54	3.32	4.36
	Maximum	5.00	3.20	4.04	4.88
	Variance	0.0486	0.0180	0.0270	0.0250

In lossless mode of JPEG2000, 5/3 reversible wavelet transform is adopted after preprocessing which includes region division, DC level shifting and reversible component transformation. The wavelet coefficients are then sent to bit plane modeling encoder and arithmetic encoder for embedded block coding with optimized truncation. In lossless mode of JPEG, the first step is linear prediction, and then the compressed data is obtained by using Huffman coding and class code. PNG mainly consists of three parts: prediction, LZ77 and Huffman coding. Table 3 illustrates the difference and comparison of soft compression and baselines. All of our methods outperform the widely-used PNG and JPEG2000 in terms of bits per sub-pixel (bpsp).

**Table 3:** Compression performance of soft compression and baselines, in bits per sub-pixel (bpsp). We emphasize the difference in percentage to soft compression for each other method in bold if soft compression outperforms the other method (RCT and DWT refer to reversible component transformation and discrete wavelet transform respectively).

Method	Prediction	RCT	DWT	Dataset			
				Malaria	BCCD	Melanoma	FIRE
Soft compression	✓	✓		2.11	2.09	2.33	1.70
JPEG (lossless mode)	✓			3.16 **+50%**	3.57 **+71%**	4.35 **+87%**	2.97 **+75%**
PNG	✓			2.83 **+34%**	3.21 **+54%**	4.15 **+78%**	2.27 **+34%**
JPEG2000 (lossless mode)		✓	✓	2.25 **+6.6%**	2.25 **+7.7%**	2.54 **+9.0%**	1.72 **+1.2%**

## Discussion

Soft compression algorithm for multi-component image makes full use of the two properties of medical images mentioned in Section I from the perspective of data mining. For the algorithm, its codebook is complete. In other words, it always contains shapes of size one, which ensures that the reconstructed image is exactly the same as the original one. Compared with the original image, the image decoded from compressed data has no information loss, which ensures the authenticity of medical images. This corresponds to the first property of medical images. In addition, soft compression algorithm uses the shape as the basic unit, reflecting the essential composition of an image. This takes advantage of the second property of medical images.

Soft compression is a universal method. It performs well even if the training stage and testing stage belong to different scenes. Soft compression algorithm is not only suitable for multi-component images, but also for single component images, because the processing of each component is independent. However, we can also consider the relationship between different components, utilizing this information to further improve the compression effect.

There are several significant differences between soft compression algorithm and other methods. These differences make soft compression more suitable and competitive to deal with medical images.

- The basic unit of soft compression is the shape, rather than the pixel.
- The location of a basic unit is no longer arranged in a definite order, but changes from a constant to a random variable.
- The codebook is no longer designed artificially or only through statistical models, but through data mining.

In the specific algorithm design, we adopt some preprocessing operations that are conducive to soft compression, such as prediction coding, mapping and layering, so that we can fully utilize the characteristics of images. The advantage of soft compression algorithm is that the codebook obtained in the training stage can be reused until it needs to be updated. When storing and transmitting images, one only needs to obtain the compressed data according to the codebook. After that, all operations can focus on the compressed data, which greatly reduce the consumption of communication and storage resources.

## Methods

### Soft compression algorithm for multi-component image

For coding, the codebook is one of the most critical things. The codebook directly determines the compression effect. The basic unit of soft compression algorithm for multi-component image is the shape. How to find the corresponding codeword of each shape is our main consideration. The codebook of soft compression is obtained by searching and dynamically updating in the dataset, which can reflect the essential information of a certain kind of images from the perspective of spatial correlation. In the process of codebook acquisition, it mainly includes prediction coding, negative-to-positive mapping, layer separation and searching.

For a multi-component image *I* that has *m* components, we first divide it into *m* single component images and perform reversible component transformation. After obtaining the new *m* components, one can process each component image independently. For each component, we will use predictive error to represent it by prediction coding^55^. Since the predictive error will have a negative value, the second step is to map it to a non-negative value, which is conducive to the subsequent layer separation operation. Layer separation is to separate the image into the shape layer and detail layer. The shape layer retains the main information of an image, which is instrumental in using the combination of locations and shapes for coding. On the other hand, the detail layer retains all the information except the shape layer. When the shape layer is obtained, search and update shape units dynamically to get the final shape set that will be used to generate the codebook. While searching in the shape layer, the distribution of intensity value in the detail layer should also be counted.

In the process of obtaining shapes, the method is to predefine a set whose elements satisfy the initial condition. During the training, the shape that meets this condition is included in the shape library. The size of the set is dynamically updated according to the frequency and weight of each shape to ensure that there is no quantity explosion. Suppose that *A* is an $M \times N$ matrix whose *i*-th row and *j*-th column are represented by vectors $\varvec{u}_{\varvec{i}}$ and $\varvec{v}_{\varvec{j}}$ respectively. The matrix whose $\varvec{u}_{\varvec{i}}$ and $\varvec{v}_{\varvec{j}}$ that follow (9) and (10) is appropriate to generate the shape.

9 $$\begin{aligned}&||\varvec{u_i}||_0 \ge {N \over 2}\quad\forall \;1\le i\le M \end{aligned}$$

10 $$\begin{aligned}&||\varvec{v_j}||_0 \ge {M \over 2}\quad\forall \;1\le j\le N \end{aligned}$$

**Figure 5:**
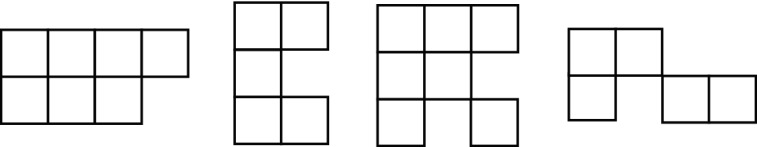
Some shapes generated with training on BCCD (these shapes are not combined with intensity values).

Removing the zero elements in the matrix and combining the remaining elements with the intensity value, one can get the shape that satisfies the initial condition. This prevents different matrices from forming the same shape. However, these shapes only become candidates, but they do not necessarily enter the codebook. In the training stage, we will match each candidate shape in the dataset. Frequency and size are the key factors to judge whether a shape can enter the codebook. We will keep the shapes with high frequency and large size. In addition, the shape with small frequency and size will be eliminated. After the final shape set is obtained, the codebook can be generated according to the size and frequency of each shape. Figure 5 shows some shapes generated with training on BCCD.

**Figure 6:**
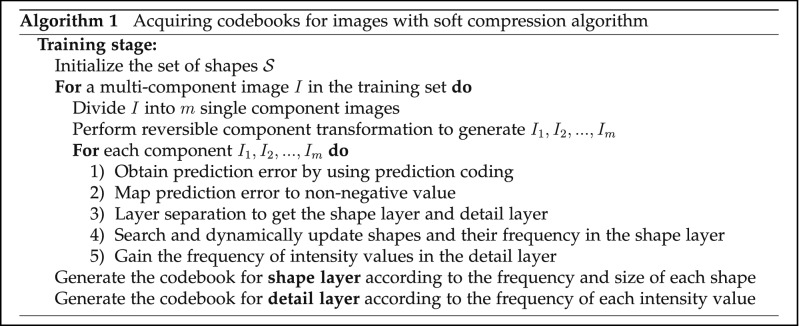
Acquiring codebooks for images with soft compression algorithm. It consists of prediction coding, mapping, layer separation, searching and assigning codewords.

For the shape layer, one needs to consider the frequency and size of each shape to generate the codebook. In this process, it aims to make the average code length as short as possible. However, for the detail layer, the optimal code can be obtained only by considering the frequency distribution of intensity values. Figure 6 illustrates the whole procedure of acquiring codebooks for images with soft compression algorithm. The codebook can be applied all the time after it is obtained, which indicates that the cost will be very tiny in the average sense. When the terminal intends to store and transmit an image, it only needs to process the compressed data, which greatly reduces the storage space and communication bandwidth.

### Encoding

**Figure 7:**
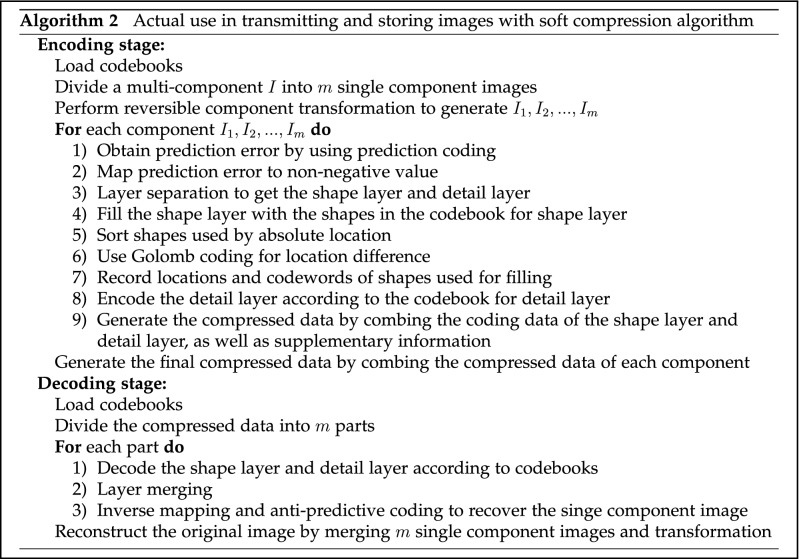
Encoding and decoding stage with soft compression algorithm for multi-component image. They will be applied after codebooks are obtained.

**Figure 8:**
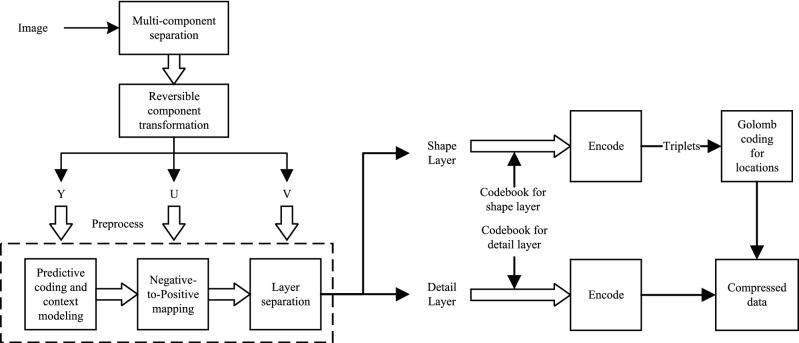
The procedure of Encoder about RGB image with soft compression algorithm for multi-component image.

The preprocessing for images of encoding is the same as the acquisition of codebooks. After a multi-component image is divided into several single component images, the prediction coding is applied for each single component image, and the predictive error is mapped into a non-negative value. The predictive error is layered to generate the shape layer and detail layer, which will be compressed by different coding methods. Figure 7 illustrates the encoding procedure. Figure 8 is the encoding process of a RGB image with soft compression.

Filling the shape layer with the codebook for shape layer yields many shapes and corresponding locations, which are represented as $(x_i,y_i,S_i)$. Since the location difference approximately obeys the geometric distribution, Golomb coding is applied for the location difference. By recording the location representation and corresponding codeword of each shape used in filling, the encoded data of shape layer can be generated. According to the codebook, the encoded data of detail layer are obtained by scanning from left to right and from top to bottom. After that, they are combined with the encoded data of shape layer and some information about an image (e.g., size) to generate the compressed file of each component. Concatenating the compressed data of each component can form the final compressed data of an image. In storage and transmission, the compressed data will be used as another lossless representation of an image.

### Decoding

The process of decoding is opposite to encoding, which is to recover the original image. It generates each component image from the compressed data through layer merging, inverse mapping, anti-predictive coding and so on. Then it can synthesize them into a multi-component image. Figure 7 illustrates the decoding part of soft compression algorithm.

### Implementation details

The algorithm is implemented by Python on a single Intel i7-9700K CPU @3.60GHz. For all datasets, the layer interface is set to 4. The batch size is set to 1 and shape degree to 0.5. The encoding an decoding complexity are both positively related to the image size and the number of shapes in codebook. The average encoding and decoding time of an image is shown in Table 4.

**Table 4:** The average encoding and decoding time of an image on different datasets with soft compression.

	Malaria (1600 × 1200)	BCCD (320 × 240)	Melanoma (512 × 512)	FIRE (2912 × 2912 )
Encoding	36.13 s	0.80 s	5.50 s	79.28s
Decoding	6.19 s	0.22 s	0.79 s	54.46s

## Conclusion

In this paper, we propose a new general representation framework for image compression. This framework takes many coding methods into account, which can be applied to represent the image compression scheme. Under the guidance of it, we design a novel coding method for medical images from the view of data mining. Soft compression algorithm for multi-component image adopts shapes as the basic unit, regarding an image as a combination of shapes. Since shapes and locations are taken into account for representing an image, the algorithm can eliminate coding redundancy and spatial redundancy at the same time. Experimental results indicate that soft compression algorithm for multi-component image can outperform the popular classical benchmarks PNG and JPEG2000.

In applications such as intelligent medicine, soft compression algorithm can help compress medical images to reduce the occupation of communication bandwidth and storage space. Of course, it can also be applied to other scenes that need lossless compression, such as precious image preservation. However, in telemedicine, the role of soft compression is not only to compress images, it may lead to more significant applications. The foreseeable research includes: (i) High fidelity video stream coding technology, which may surpass the current international standards. (ii) Efficient channel coding technology suitable for certain types of images, which is based on shapes rather than pixels. (iii) Develop the corresponding storage coding and fast encoding and decoding methods, as well as local image information extraction methods. (iv) Combined with artificial intelligence, one can develop a widely used software platform and open source library.

In the future, on the one hand, it can improve the performance of the algorithm by taking advantage of the characteristics of medical images to do the corresponding preprocessing. On the other hand, mining more effective shape acquisition methods can bring better results. In addition, the combination of soft compression algorithm and other coding methods such as transform domain, can achieve efficient lossy compression.

## Data Availability

The code used and the datasets analyzed during the current study are available from the corresponding author on reasonable request and can also be found at https://github.com/ten22one/Soft-compression-algorithm-for-multi-component-image
